# Comparative fungal diversity and dynamics in plant compartments at different developmental stages under root-zone restricted grapevines

**DOI:** 10.1186/s12866-021-02376-y

**Published:** 2021-11-16

**Authors:** Muhammad Salman Zahid, Dongmei Li, Hafiz Umer Javed, Irfan Ali Sabir, Lei Wang, Songtao Jiu, Shiren Song, Chao Ma, Dapeng Wang, Caixi Zhang, Xuhui Zhou, Wenping Xu, Shiping Wang

**Affiliations:** 1 Department of Plant Science, School of Agriculture and Biology, Shanghai Jiao Tong University, Shanghai, 200240 China; 2 Shanghai Key Laboratory for Urban Ecological Processes and Eco-Restoration, School of Ecological and Environmental Sciences, East China Normal University, Shanghai, 200241 China

**Keywords:** Grapevine, Root-zone restriction, Fungal diversity, Fruit quality, Rhizosphere, Phyllosphere

## Abstract

**Background:**

The root-zone restriction cultivation technique is used to achieve superior fruit quality at the cost of limited vegetative and enhanced reproductive development of grapevines. Fungal interactions and diversity in grapevines are well established; however, our knowledge about fungal diversity under the root-zone restriction technique is still unexplored. To provide insights into the role of mycobiota in the regulation of growth and fruit quality of grapevine under root-zone restriction, DNA from rhizosphere and plant compartments, including white roots (new roots), leaves, flowers, and berries of root-zone restricted (treatment) and conventionally grown plants (control), was extracted at three growth stages (full bloom, veraison, and maturity).

**Results:**

Diversity analysis based on the ITS1 region was performed using QIIME2. We observed that the root-zone restriction technique primarily affected the fungal communities of the soil and plant compartments at different growth stages. Interestingly, *Fusarium, Ilyonectria, Cladosporium* and *Aspergillus spp* observed in the rhizosphere overlapped with the phyllosphere at all phenological stages, having distinctive abundance in grapevine habitats. Peak richness and diversity were observed in the rhizosphere at the full bloom stage of control plants, white roots at the veraison stage of treatment, leaves at the maturity stage of treatment, flowers at the full bloom stage and berries at the veraison stage of control plants. Except for white roots, the diversity of soil and plant compartments of treated plants tended to increase until maturity. At the maturity stage of the treated and control plants, the abundance of *Aspergillus spp.* was 25.99 and 29.48%, respectively. Moreover, the total soluble sugar content of berries was 19.03 ^o^brix and 16 ^o^brix in treated and control plants, respectively, at the maturity stage.

**Conclusions:**

This is the first elucidative study targeting the fungal diversity of conventional and root-restricted cultivation techniques in a single vineyard. Species richness and diversity are affected by stressful cultivation known as root zone restriction. There is an association between the abundance of *Aspergillus spp.* and fruit quality because despite causing stress to the grapevine, superior quality of fruit is retrieved in root-zone restricted plants.

**Supplementary Information:**

The online version contains supplementary material available at 10.1186/s12866-021-02376-y.

## Background

Endophytes and epiphytes significantly modulate vine vigour, development, and yield by colonizing grapevines (*Vitis vinifera*) [1–4]. The surrounding environment, such as soil, air, precipitation, animal vectors, and natural forests, plays a vital role in raising microbes, which subsequently colonize epiphytes and endophytes [5]. The plant proactively recruits microbes by releasing exudates from their roots [6, 7]. These microbes can be further transferred to berries, thus profoundly impacting berry quality [8, 9].

Recent developments have indicated the inevitable role of microbes that are structured and assembled in the geographical locality towards shaping the terroir of grapevines and additionally having an impact on defining the core soil mycobiome [10]. On a scale of hundreds to thousands of kilometres, biogeographic patterns are generally detected [11], and multiple elements, including climate and topography, soil qualities, and even local anthropogenic practices, influence the outcome [4, 12, 13]. The majority of microbial spatial pattern investigations in grapevines have been performed at large scales and focused on understanding communities present in the rhizosphere, roots, bark, grapes, leaves and must [5, 14, 15]. However, the diversity and importance of microbial ecological variation within a vineyard under two different cultivation methods (conventional and root-zone restriction) have still not been investigated.

The composition of mycobiomes is contingent on numerous variables, including the host plant and its density, availability of nutrients, environmental conditions, and interactions with surrounding microorganisms [16]. The reported confirmation of the major regulatory elements has been equivocal. Distinct habitats and geographical scales have different soil microbiome spatial regulators [17]. Organ-associated microbiota are derived mainly from soil, and the migration of microorganisms from soil can potentially affect the regional patterns in wine chemosensory properties [18]. Fungal communities are affected by soil-plant compartments in their diversity, composition and functionality [19]. Under normal growth conditions, endophytes can have impartial or adverse effects on the host plant, while they can be beneficial under more severe conditions or during various phenological stages [20]. Fungal communities across the soil and multiple plant compartments under a stressful environment named the root-zone restriction technique have not been thoroughly investigated. The composition of the microbiome does not remain constant throughout time, as in other plant systems, but changes in response to plant development [21, 22] and has a significant impact on plant health and production.

With the advancement of high-density plantation methods, the root-zone restriction technique is also being applied widely and practised in fruit tree cultivation with various advantages. Due to this system, the roots are physically confined in a specific space, thus regulating vegetative and reproductive growth and improving fruit quality, and more plants can be planted in a given area. Several studies on grapes [23–25], cherry [26], carambola [27] and apple [28] have reported altered plant architecture.

The main objective of this technique is to have superior fruit quality for consumers. Numerous aspects of root-zone restriction techniques have been reported, such as increased root mass and the number of fibrous roots, reduced shoot growth, and improved fruit set and fruit quality in grapevines [29]. C6 compounds and terpenes were the main free and bound volatile compounds of Shine Muscat grapevine [30]. Increased root hair development [31], increased anthocyanin accumulation in berries [32], and twenty-three novel grapevine-specific miRNAs were involved in root formation [23] and nitrogen metabolism [33]. Nevertheless, many aspects have yet to be explored, and among these aspects, the microbiological aspect is of vital importance, which aids us in obtaining a clearer perception of the microbes involved in this technique. However, at present, the consequences of root-zone restriction on the associated fungal microbiota are unexplored.

In this study, we sampled the fungal communities associated with Muscat Hamburg grapevines grown in the same vineyard under two different growing techniques, i.e., conventional and root-zone restriction cultivation to provide intra-vineyard variability fungal communities. We hypothesized that plant roots influence the recruitment of the microbiota, so under root-zone restriction, the root architecture is altered, and as a result, microbial recruitment and fungal diversity in the plant compartments will be affected accordingly. High-throughput amplicon sequencing of the fungal ITS1 region was performed to unravel the effect of the root-zone restriction technique on the fungal diversity and composition of conventionally grown grapevines (intra-vineyard variability), grapevine habitats (rhizosphere, roots, leaves, flower and berries) and grapevine phenological stages (full bloom, veraison and maturity) along with physiological parameters and fruit quality. Our study comprehensively characterized the mycobiome of grapevine compartments across seasons and root-zone restriction.

## Results

### Vine growth and berry quality under root-zone restriction

Our treatment (root-zone restriction) had a significant effect on the trunk diameter and chlorophyll (Table 1). The chlorophyll and trunk diameters were significantly higher in the root-restricted grape plants than in the control. Regarding cane diameter and shoot length, the treatments showed a statistically similar effect.

**Table 1:** Effect of treatments and growth stages on the morphological parameters

Development stage	Cane diameter		Trunk Diameter		Chlorophyll content		Shoot length	
	Treatment	Control	Treatment	Control	Treatment	Control	Treatment	Control
**Full bloom**	24.53 ± 0.79b	25.08 ± 1.03b	27.14 ± 1.61c	36.36 ± 1.01b	33.61 ± 0.53d	31.13 ± 0.79e	86.90 ± 4.3c	78.03 ± 6.7c
**Veraison**	30.63 ± 0.77a	30.06 ± 1.68a	34.71 ± 1.97b	39.80 ± 0.88ab	40.45 ± 0.77bc	39.76 ± 0.47c	179.60 ± 3.1b	189.03 ± 6.7b
**Maturity**	31.83 ± 1.69a	31.63 ± 1.53a	36.96 ± 0.36b	42.80 ± 1.8a	42.60 ± 0.67a	41.80 ± 1.2ab	256.27 ± 2.3a	265.03 ± 6.7a

Mean ± standard deviation (*n* = 3) of the same parameters followed by different letters are significantly different (*p* < 0.05)

**Fig. 1:**
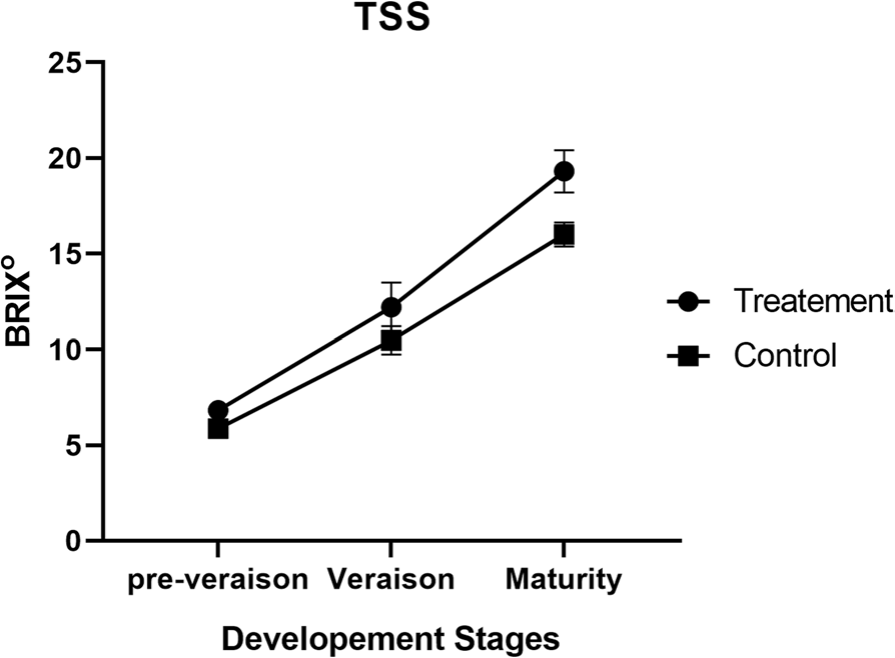
TSS (Brix) figure at three developmental stages and increasing in the treatment plants compared to the control

Total soluble solids (TSS), an indicator of fruit quality, showed remarkable significance between treatment and control plants. The fruit harvested from root-restricted grape plants exhibited a higher level of TSS than the control and increased significantly with changes in phenological stages (veraison and maturity). At the pre-veraison stage, the TSS content was higher in the treatment group than in the control group, but the difference was not statistically significant, as shown in Fig. 1 and Additional file 12.

Phenological stages significantly affected all growth parameters (cane diameter, trunk diameter, chlorophyll content, and shoot length). As the phenological stages changed from full bloom to maturity, the chlorophyll content and shoot length rose considerably. The cane diameter and shoot length remained higher in the treatment, but statistically, they were homogenous to each other.

### Intra-vineyard variation among the fungal communities of soil and plant compartments

Microbiome bioinformatics, including normalization, was performed with QIIME2 2019.4 [34] with slight modification according to the official tutorials (https://docs.qiime2.org/2019.4/tutorials/). A total of 1,939,851 quality filtered fungal ITS sequences were generated after paired-end alignments, quality filtering, and deletion of chimeric, singleton, mitochondrial, and chloroplast sequences. The average length of the sequences was 251 bp (Additional file 2), and samples were rarefied to 49,138 sequences per sample. The ASVs obtained were 7890 with an average of 264 per sample and with a threshold of 97% pairwise identity (Additional file 11). A petal map was constructed to identify the shared and distinctive ASVs between the rhizosphere, white roots, leaves, flowers, and berries. Only 3 ASVs were shared across all the samples and growth stages in both cultivation methods. (Additional file 3).

### Rhizosphere

In the rhizosphere (S), Additional file 1, a significant chunk of 2873 ASVs (36.41%) was conceived, along with 99 shared ASVs. A higher number of ASVs was recorded in our treated (root-zone restricted) plant in the veraison soil treatment (VST) and maturity soil treatment (MST), and fewer ASVs were recorded in the full bloom soil treatment (FBST) than in the veraison soil control (VSC), maturity soil control (MSC) and full bloom soil control (FBSC).

### White roots

White Roots (WRs) Additional file 1 contained 1696 unique and 20 shared ASVs (21.49%) among all the samples*.* Numbers remained low in the full bloom white root treatment (FBWRT) and higher in the veraison white root treatment (VWRT) and maturity white root treatment (MWRT) samples than in the full bloom white root control (FBWRC), veraison white root control (VWRC), and maturity white root control (MWRC) samples.

### Leaves

In total, 1473 ASVs were found in leaves, which accounted for 18.66% of the total, and 19 ASVs were shared across all the leaf samples (Additional file 1). Full bloom leaf treatment (FBLT) stood at a low ASV count compared to veraison leaf treatment (VLT) and maturity leaf treatment (MLT) regarding full bloom leaf control (FBLC), veraison leaf control (V_L_C), and maturity leaf control (MLC), respectively.

### Flower/berry

Flower had a total of 624 ASVs, accounting for 7.90% of the total amplicon sequence variants. The full bloom flower treatment (FBBT) was low compared to the full bloom flower control (FBBC). In the berries, the total count was 1029 (13.04%). The berries stood lower in veraison berry treatment (VBT), higher in maturity berry treatment (MBT) than veraison berry control (VBC) and maturity berry control (MBC), respectively, along with 33 shared ASVs in Additional file 1. For detailed ASV count, Additional files 4 and 11.

### Fungal community structures of soil and plant compartments

**Fig. 2:**
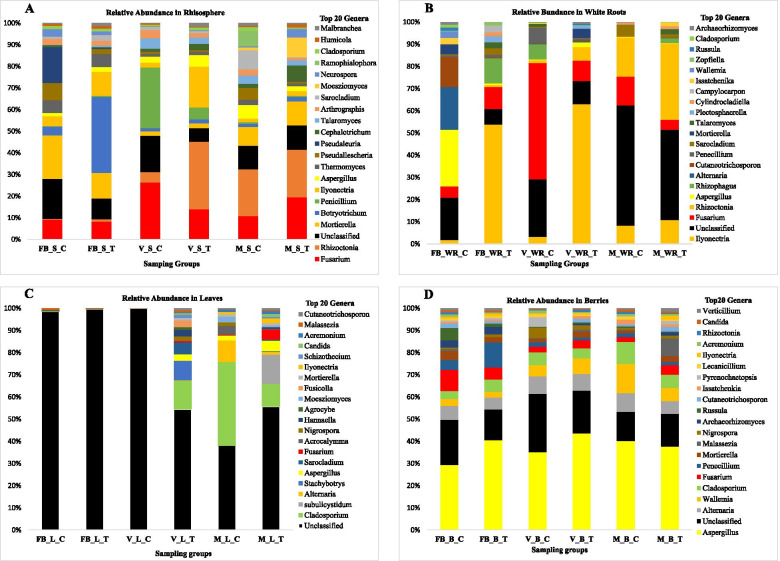
Histogram showing the relative abundance of the top 20 fungal genera recorded from the different rhizosphere (**A**), white root (**B**), leaf (**C**) and berry (**D**) groups at three phenological stages of Full Bloom (FB), Veraison (V) and Maturity (M)

*Ascomycota* was the most abundant phylum present in grapevine habitats, followed by *Basidiomycota, Mortierellomycota and Glomeromycota* (Additional file 5). The abundant fungal genera were *Fusarium, Aspergillus, Ilyonectria, Cladosporium, Alternaria, Wallemia and Rhizoctonia,* having relative abundances above 1% across all the samples. *Fusarium* was most abundant in the rhizosphere in VSC (18.38%), *Ilyonectria* in white roots in VWRT (44.99%), *Cladosporium* in leaves in MLC (32.09%) and *Aspergillus* in berries MBC (29.48%) (Fig. 2).

### Diversity of fungal communities associated with soil and plant compartments at different phenological stages

**Fig. 3:**
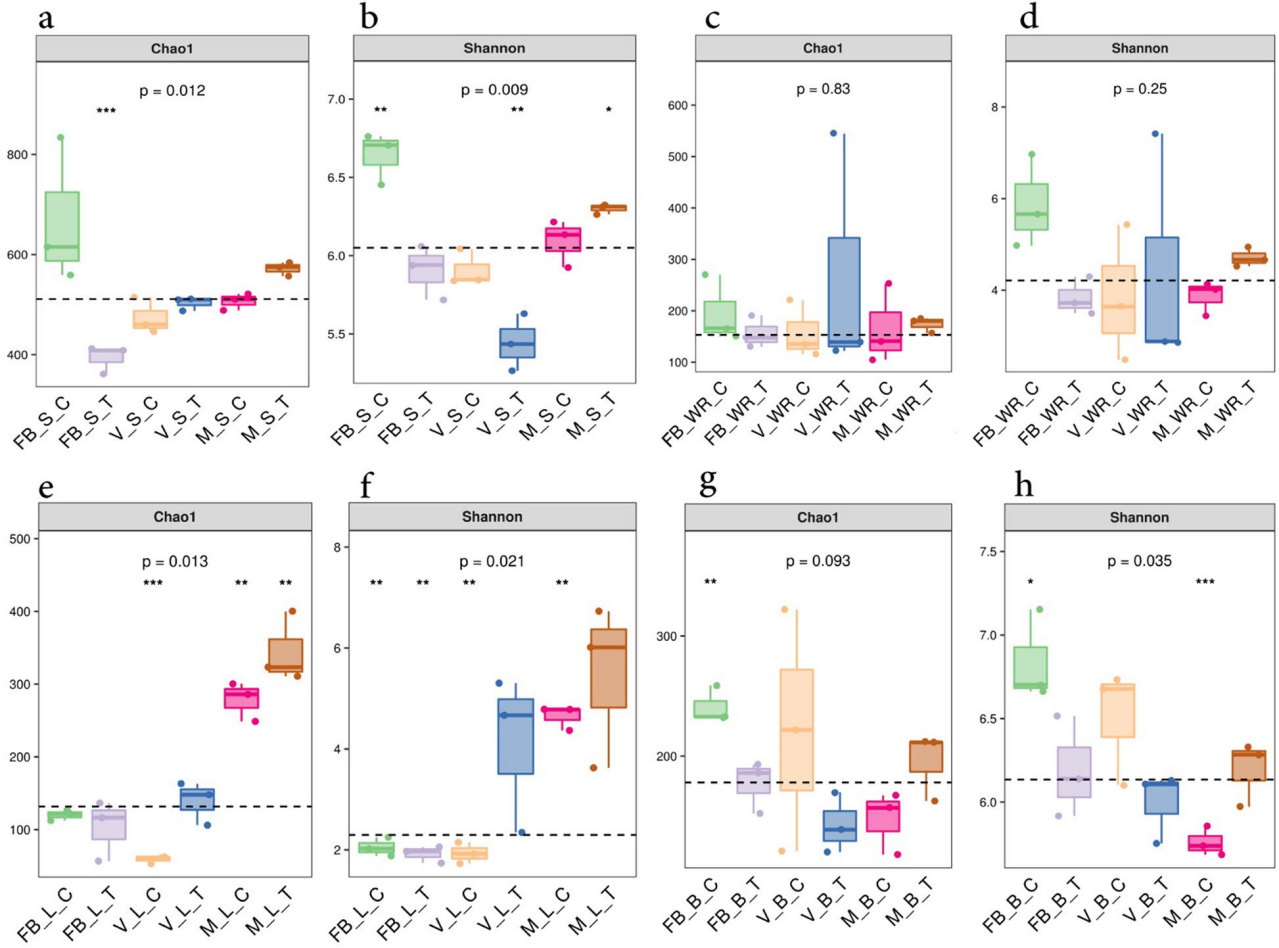
Box plot showing the Chao1 and Shannon indices used to explore the alpha diversity in our samples of rhizosphere (**a**), white roots (**b**), leaves (**C**) and berry (**d**) at three growth stages, full bloom (FB), veraison (V) and maturity (M), with comparison to treatment (T) and the control (C)

Alpha diversity indices were exercised to explore the fungal diversity of the rhizosphere, which showed the difference between the treatment and control groups at three main phenological stages. Comparatively, less diversity and richness were found in our treated samples (Shannon Chao1 indices) at the veraison stage (Fig. 3A, B). White roots of treated plants were more diversified and richer at the veraison stage (VWRT) than control plants. Slightly higher values of the Shannon index were obtained at the maturity stage (MWRT), as shown in Fig. 3C, D.

A considerable abundance of unclassified fungal communities was recorded in the leaf samples at FBLC, FBLT, and VLC, unlike the trend of maximum diversity and richness at the maturity stage in the treated plant leaves (MLT) that was recorded (Fig. 3E, F). Following the same inclination of treatment deficient in richness and diversity, our flowers (FBBT) of the treated plants were the same. The FBBT stood slightly low, according to Shannon, as shown in Fig. 3G, H, compared to the control flowers. The berry sample of treated plants at the veraison (VBT) stage was low and at maturity (MBT) was high in terms of richness and diversity of fungal communities (Fig. 3G, H). Species accumulation and rank abundance curves were formulated to track the increase in species richness in the population as the sample size increased and the abundance of ASVs obtained from the study (Additional files 6 and 7).

**Fig. 4:**
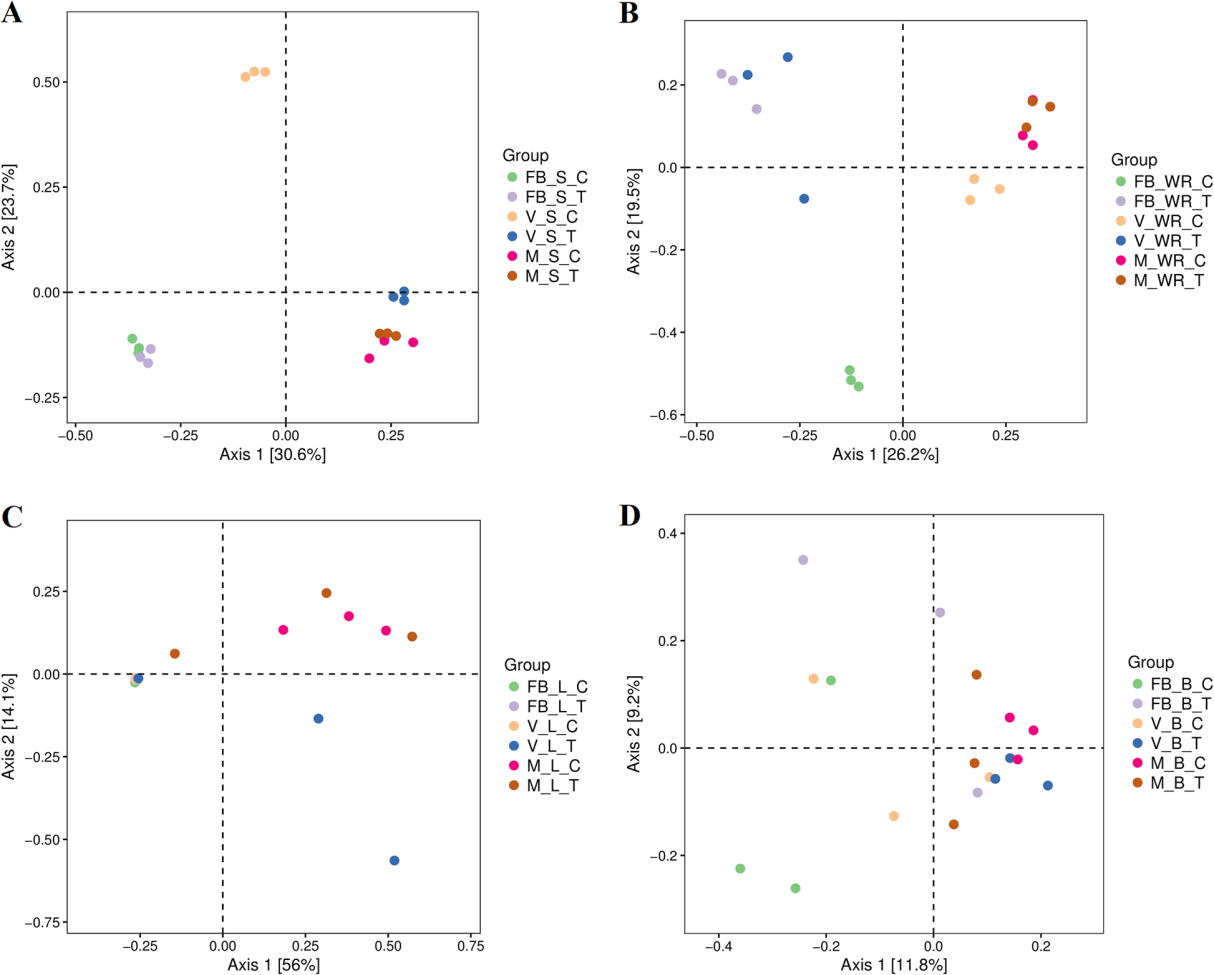
Principal coordinate analysis (PCoA) (two-dimensional plot Axis1 vs. Axis2). Based on Bray-Curtis distance used for exploring beta diversity between treated (T) and control (C) plants at full bloom (FB), veraison (V) and maturity (M). Rhizosphere (**a**), white roots (**b**), leaves (**c**), berry (**d**)

Bray-Curtis distance metrics were used to explore the beta diversity and represented classical multidimensional scaling as PCoA and UPGMA. The maximum distance was recorded between the soils of treated and control plants VST and VSC at the veraison stage (Fig. 4A). White roots of FBWRC and FBWRT differed from each other, followed by VWRC and VWRT (Fig. 4B). The next plant organ under consideration is the leaves. Despite having the majority taxa as unclassified at FBLC, FBLT and VLC, we found that leaves of the treated plants at the veraison stage VLT were more different from the rest of the others. At the same time, no significant difference was recorded between the leaves at the maturity stage of treated (MLT) and control (MLC) plants (Fig. 4C). Flowers at FBBC and FBBT were apart, followed by berries at the veraison and maturity stages, as shown in Fig. 4D.

Permanova analysis was performed using the Adonis function in QIIME2 for each plant organ separately to check the variation between the groups and within the groups based on Bray-Curtis dissimilarity distances, while pseudo-F was the test statistic. The value of pseudo-F, P and R^2^ for soil was 18.9532, 0.001 and 0.88, for white roots it was 4.9761, 0.001 and, 0.67 for leaves 3.15223, 0.002 and 0.56 and for berry, the values were 1.20633, 0.003 and 0.33, respectively. The detailed table for permanova (Adonis) is found in Additional file 13. Significance was higher between the groups than among the groups. Plant organs as a whole were more significant than the individual plant organs sampled at different growth stages. Clustering analysis was performed to analyse the similarity between the samples. UPGMA grounded on the Bray-Curtis distance matrix was used (Additional file 8), which showed the similarity between our samples. Our treatment and control samples were largely nonsimilar, depicting diversity.

### Drivers of diversity in sample groups

**Fig. 5:**
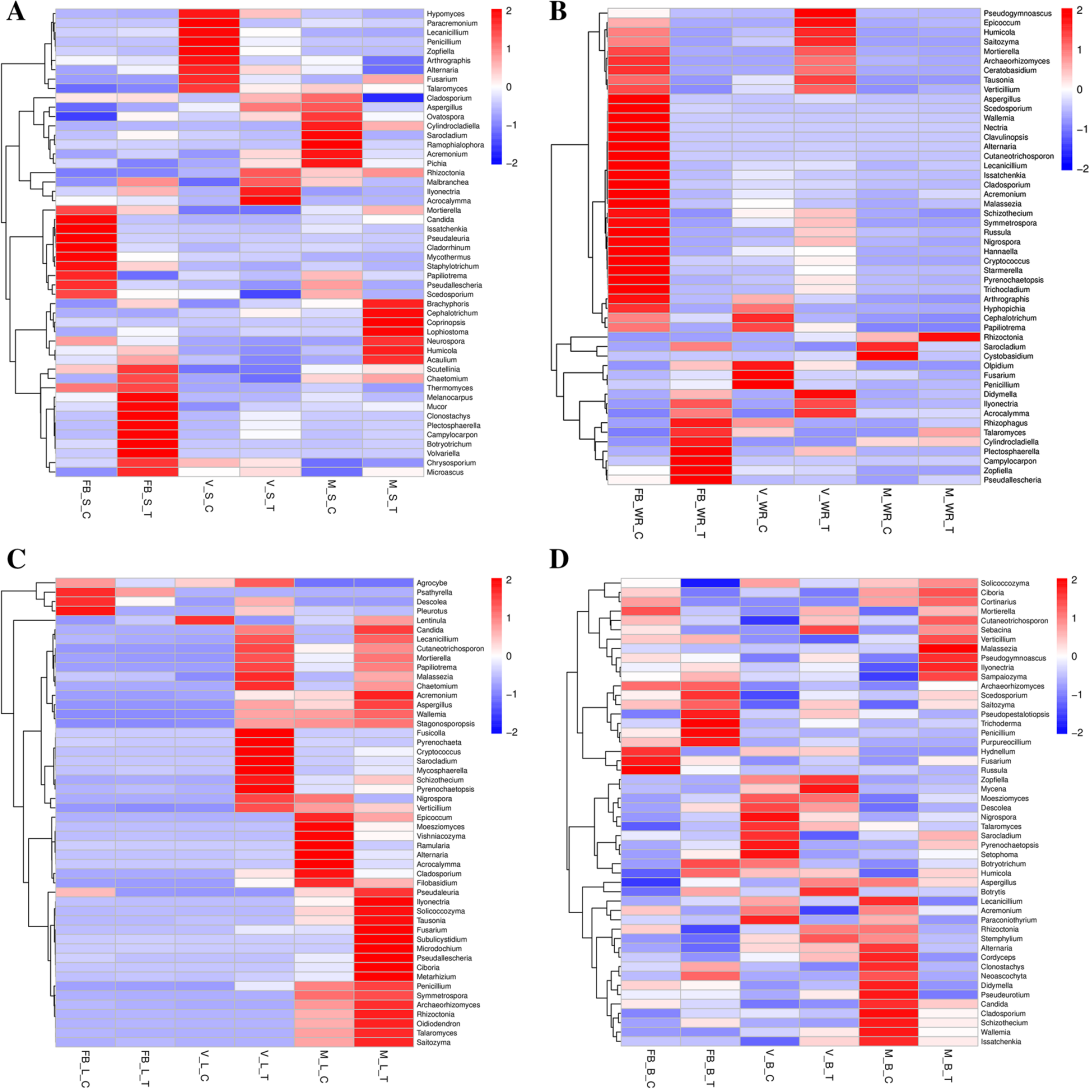
Combined heat level map of the community composition with cluster analysis. Rhizosphere (**a**), white roots (**b**), leaves (**c**), berries (**d**). Sample groups are clustered according to the similarity index on abscissas, while the ordinate has different identified species

A heat map was visualized using the abundance data of the top 50 most abundant genera. The most discriminant taxa and their correlation with the respective sampling groups are shown in Fig. 5. UPGMA performed clustering according to Euclidean distance for samples and Pearson correlation coefficient for clustering of species. Differential species in the soil, e.g., *Scutellina,* were most abundant, with maximum abundance in sample FBST, while in FBSC, the abundance was near 0, as shown in Fig. 5A. Every sampling group had distinctive species, which elucidates broad diversity among the identified species. Similarly, Fig. 5B demonstrates the fungal communities associated with white roots, leaves in Fig. 5C, and unique fungal taxa linked with flowers/berries in Fig. 5D.

**Fig. 6:**
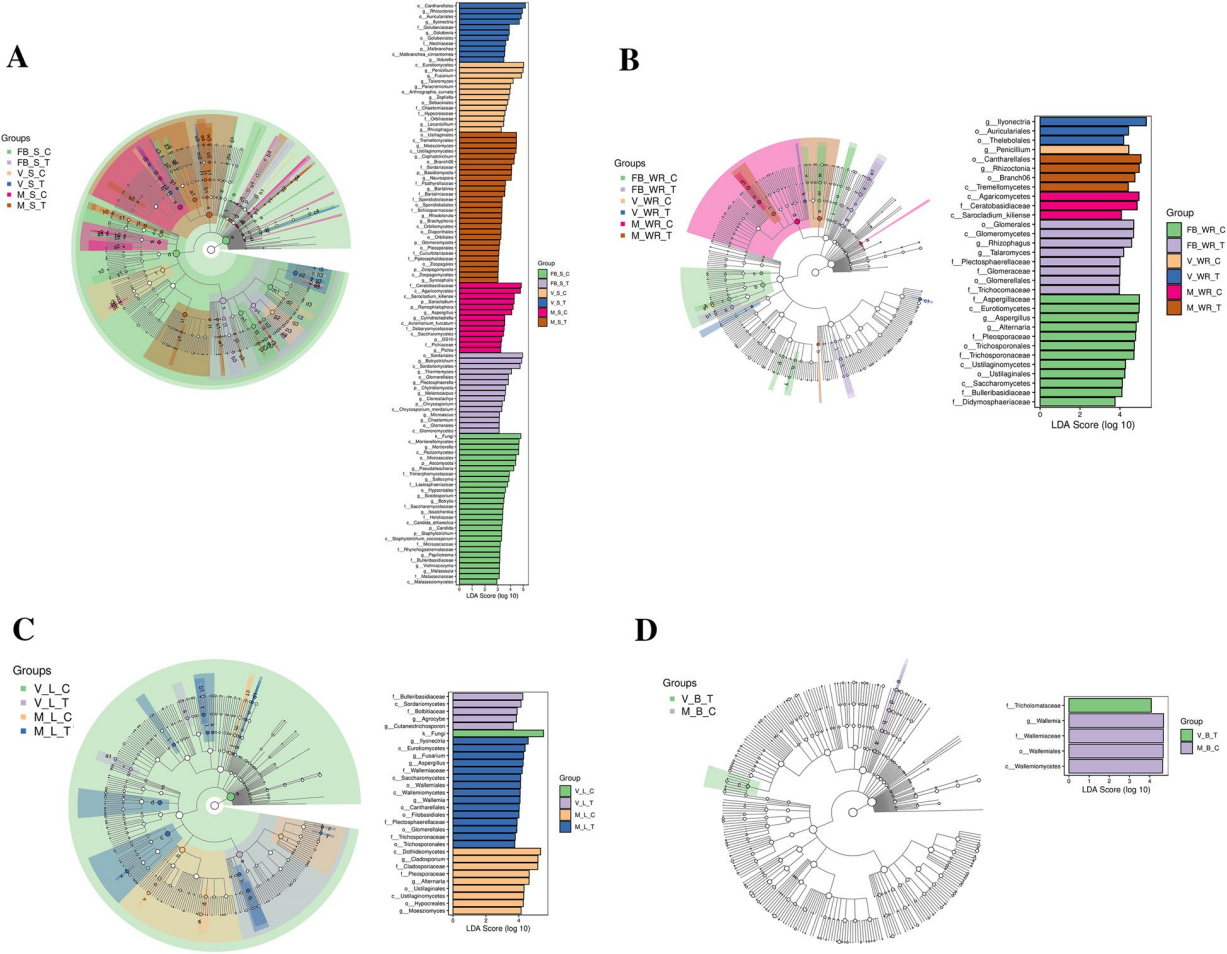
LEfSe (linear discriminant analysis (LDA) effect size) for taxa identification between the treatment and control plant groups. In each portion of the above figure, the cladogram and its corresponding histogram of LDA scores are shown. The cladogram (Wilcoxon *p* value < 0.05) illustrates taxa with significantly different abundances. Coloured dots from the centre to outwards signify the kingdom system in chronological order. Hollow nodes represent insignificant differences, while the coloured nodes indicate significant differences between groups. Classification units in the histogram are sorted according to the score value (LDA effect size > 2) to describe their specificity in the sample grouping. LEfSe for rhizosphere (**a**), white roots (**b**), leaves (**c**) and flowers/berries (**d**) at full bloom (Fb), veraison (V) and maturity (M) of control (C) and treated (T) plants

LEfSe (LDA effect size) is an analysis method that combines the nonparametric Kruskal-Wallis and Wilcoxon rank-sum tests with linear discriminant analysis (LDA) effect size. An LDA score of a minimum of 2 was used to identify different bacterial groups with statistical significance (*p* < 0.05). The LDA score reveals distinctive microbial taxa distribution among the samples. Cladograms for a respective sample of soil, white roots, leaves, flowers, and berries were illustrated, along with the hierarchical relationship from phylum to genus. The phylum *Ascomycota* (*p* = 0.01, LDA score = 4.39) was significant in FBSC, *Basidiomycota* (*p* = 0.01, LDA score = 4.09) in MST and order *Sordariomycetes* (*p* = 0.01, LDA score = 4.74) in FBST, and they predominantly occupied the microbial taxa of soil samples, as shown in Fig. 6A. White roots of the grapevine exhibited class *Agaricomycetes* (*p* = 0.04, LDA score = 4.95) in MWRC, class *Eurotiomycetes* (*p* = 0.01, LDA score = 4.96) in FBWRC and class *Glomeromycetes* (*p* = 0.01, LDA score = 4.68) in Fig. 6B. The majority of observed fungal taxa in the leaves of both control and treated plants were unclassified fungi, although we established class *Eurotiomycetes* (*p* = 0.01, LDA score = 4.38) in MLT, class *Dothideomycetes* (*p* = 0.01, LDA score = 5.33) in MLC, and class *Sordariomycetes* (*p* = 0.01, LDA score = 4.14) in VLT in abundance in the leaves (Fig. 6C). The most significant taxa obtained in the berry according to LefSe were family *Tricholomataceae* (*p* = 0.03, LDA score = 4.07) in VBT and family *Wallemiaceae* (*p* = 0.03, LDA score = 4.67) in MBC (Fig. 6D). Detailed taxa according to the cladogram are shown in Additional file 9.

**Fig. 7:**
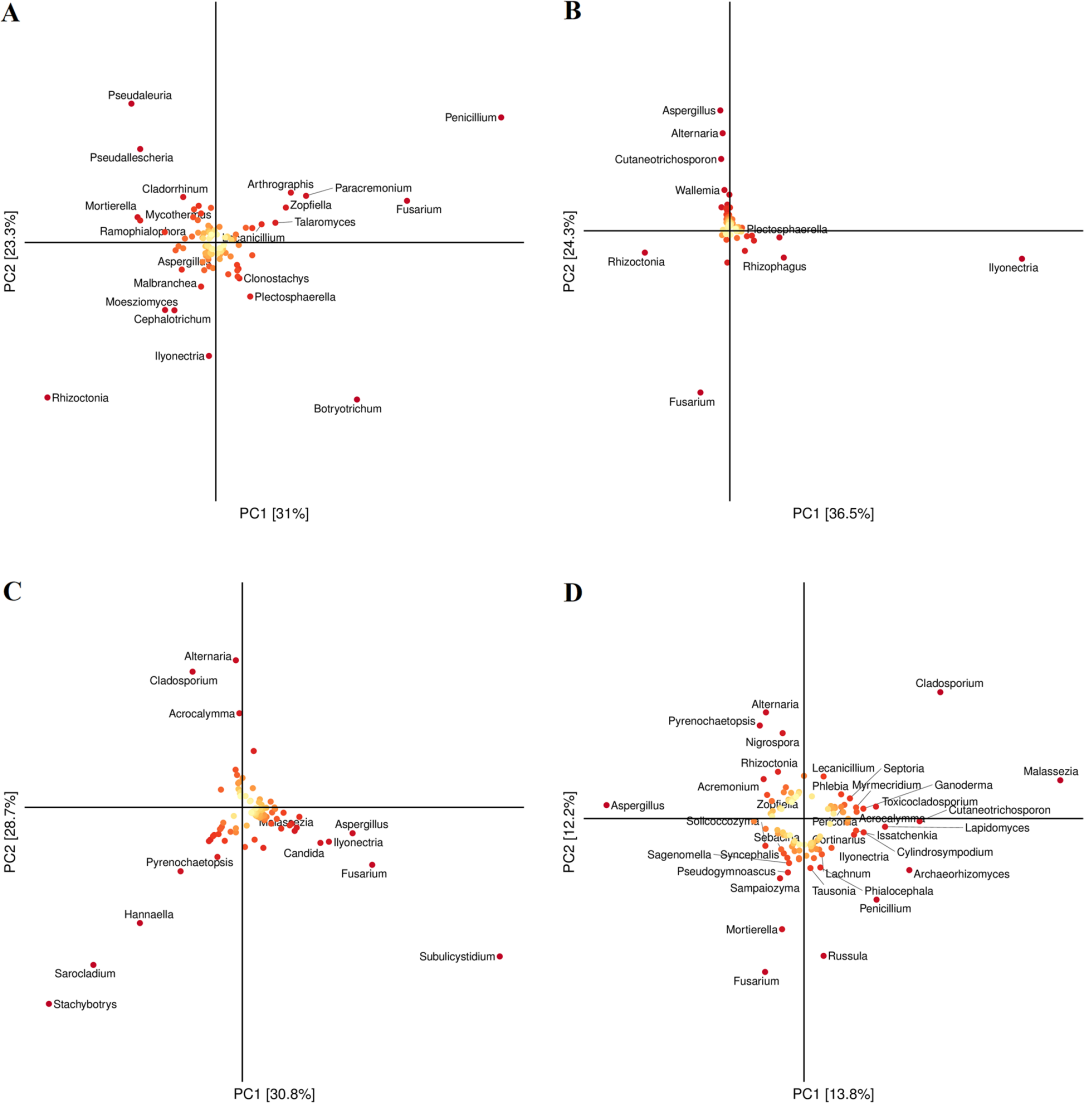
Orthogonal partial least squares discriminant analysis (OPLS-DA) of determinants in positive and negative. Each point on the picture represents a species (genus by default). According to their VIP values of the species (variable importance in projection). The colour is from yellow to red to indicate the value from small to large. The physical unit length ratio of the two coordinate axes is set to 1 by default (different from the interpretation ratio). OPLS-DA for rhizosphere (**a**) and white roots (**b**) leaves (**c**) and flowers/berries (**d**) at full bloom (Fb), veraison (V) and maturity (M) of control (C) and treated (T) plants

According to OPLS-DA (orthogonal partial least squares discriminant analysis), the main taxa responsible for the variation were identified with reference to their groups. Rhizosphere (Fig. 7A). FBST was on the positive side of PC1 and negative of PC2, while MSC was on the positive side of both PC1 and PC2. *Penicillium* had a score of 0.51 along PC1 and 0.29 on PC2. *Rhizoctonia* − 0.30 and − 0.37 and *Botryotrichum* 0.25 and − 0.37 are shown along PC1 and PC2, respectively. Similarly, in white roots (Fig. 7B), MWRT and FBWRT remained on the positive side of the PC, and VWRT stood almost on the equatorial line between both axes. The unique species found were *Ilyonectria, which* scored 0.72 on the positive side of PC1 and − 0.10 on the negative side of PC2. *Fusarium* remained on the negative sides of both axes with scores of − 0.07 and − 0.59 on PC1 and PC2, respectively. *Rhizoctonia* remained at the negative sides of both coordinates, with − 0.20 on PC1 and − 0.08 on PC2. Considering the leaves, VLT remained on the positive side of PC1, and VLC remained on the positive side of PC2. A notable genus in the leaves, according to discriminative analysis, was *Subulicystidium at* 0.52 and − 0.32 in PC1 and PC2, respectively. *Stachybotrys* was − 0.39 and − 0.43 along PC1 and PC2, respectively, as shown in Fig. 7C. Groups of the sampled berries showed much variation. VBT covered three sides of coordinates, FBBC and FBBT remained on the positive side of PC1, and MBC and MBT remained on the positive side of PC2. Prominent genus VIP scores were *Malassezia* 0.51 and 0.08*, Cladosporium* 0.27 and 0.28, *Alternaria* − 0.07 and 0.24*, Aspergillus* − 0.39 and 0.03*,* and *Fusarium spp* − 0.07 and − 0.34 alongside PC1 and PC2, respectively (Fig. 7D).

## Discussion

We characterized the fungal diversity in multiple grapevine parts at three major phenological stages: full bloom, veraison and maturity, grown under the root-zone restriction technique. Soil microbes have a role in plant development and disease prevention [35–37]. Soil microbial communities are essential in the cultivation of grapes, as it has been shown that soil microbiota serve as a reservoir of grape-colonizing microorganisms [38], contributing to the shaping of regional wine traits [8]. The rhizosphere and every plant organ responded differently according to its environment and growth stage. The rhizosphere of treated plants was less diverse at the full bloom (Shannon = 5.86) and veraison stages (Shannon = 5.43), as reported [5], while the rhizosphere of treated plants was more diverse at the maturity stage (Shannon = 6.29), which contradicted the prior cited work. White roots of treated plants were more diverse at the veraison (Shannon = 4.38) and maturity stages (Shannon = 4.69) than control plants. Here, the trend of our study contradicts the study cited [5], as the authors mentioned a decline in diversity at veraison and then an increase at the maturity stage. Considering that the root-zone restriction technique alters the root architecture of the plant, it became denser than usual. We suggest that the diversity between the rhizosphere and roots is related because root microbes are derived from the rhizosphere [14], and thus, both root architecture and root exudation shape the rhizosphere microbiota [39]. Vines grown under root-zone restriction with altered root architecture presumably recruit specific fungal species residing around the rhizosphere at different phenological stages to aid them in performing different tasks. Targeted studies to explore the signalling mechanism between fungi and roots are indispensable.

Our results of leaf fungal diversity contradicted [5]. According to the authors of [5], leaf fungal diversity showed a decline at the maturity stage, but the leaves of our root-zone restricted plant exhibited greater diversity at the veraison (Shannon = 4.08) and maturity stages (Shannon = 5.45), which could be due to the effect of migration, colonization, persistence, and community succession as a key factor driving the phyllosphere microbial community [21].

It is essential to mention that we obtained the majority of unclassified fungal taxa at FBLC, FBLT, and VLC. We amplified the ITS1 region, and it has limitations as described [40]. The lack of consensus in the lineage-specific cutoff value for species determination makes it challenging to classify morphologically related cryptic species using the ITS regions correctly. There is disagreement in using ITS1 or ITS2 as a barcode and which ITS primer sets have the best resolution for fungal diversity [41].

The fungal diversity of berries increased from full bloom to maturity in our treated plant compared to the control (Fig. 3H). Sugar build up and exudation as ripening progresses might reveal this result. The accumulation of carbohydrates promotes fungal colonization, according to scientific evidence [42, 43]. We obtained a higher value of TSS at maturity, which could be due to the presence of fungi in grape berries [44]. *Aspergillus spp.* were most abundant, followed by *Alternaria spp.* in the berries. The higher value of TSS in the berries could be due to *Aspergillus* or might be a result of a synergetic effect of both. The endophytic activity of both of them has been reported in numerous studies. Endophytic *Aspergillus* species have proven their ability to produce many active secondary metabolites [45]. *Aspergillus flavus*-inoculated soybean and sunflower seedlings had higher amounts of soluble sugars, total proteins and total lipids than the control seedlings of both species [46].

Similarly, an isolate of *A. niger*, SkNAn5, resulted in a significant increase in the total phenolic content, salicylic acid in roots and shoots, chlorophyll content and Brix index of tomato [47]. C2J6, a strain of *Aspergillus niger,* was reported to produce stable high resveratrol production. *Alternaria spp*. were also reported to produce novel metabolites in grapevine berries [48].

Fungal endophytes could have a constructive influence on the increase in trunk diameter and chlorophyll content of root-restricted plants, as observed here. Some of these macronutrients (e.g., N and Mg) are critical constituents of chlorophyll, so arbuscular mycorrhizal fungi might have increased the nutrient content in our treated plants and subsequently resulted in higher chlorophyll content [49–51].

Cambium growth is subjected to indole-3-acetic acid (IAA) production and regulation [52]. *Tricholoma* and *Aspergillus spp.* are responsible for IAA production [53, 54], considering that both of these occur in our metagenome sequences of treated plants. Some reports have established that root-zone restriction decreases the nitrogen concentration in the shoots, flower clusters, trunks and canes of grapevines [33], leaves of euonymus [55] and peach [56], which could be the reason for decreased vegetative growth in our treated plants. Fungal diversity is also regulated by nitrogen content in plants, as reported by [57], so this could be a reason because we found maximum diversity in white roots and leaves at the veraison and maturity stages, as some ectomycorrhizal fungi are responsible for increasing the nitrogen content in wheat plants [58].

The temporal transition between the plant’s organs at different phenological stages was carried out throughout. *Ascomycota* remained dominant at full bloom and *Basidiomycota* at veraison and maturity in the rhizosphere and white roots, but leaves, flowers, and berries did not follow this trend, possibly because the richness patterns of both phyla differ from each other, which needs further investigation, or fluctuations could be due to some hidden driving factors to regulate plant-microbe communication. Unlike bacteria, fungal populations are driven primarily by biogeographic patterns, climate, location, soil chemistry and spatial patterns [13, 59]. Plant species, spatial locality, plant growth development, leaf structure, chemical composition, and secretion affect phyllosphere microbial structure [60, 61].

Our samples of treated plants were clustered closely based on the Bray-Curtis distance matrix compared to the control plants, showing the variation and distance. We observed that core microbes varied along the growth stages [5]. Shared ASVs were few throughout our samples, and the remainder were separated and dissimilar from one another. The maximum shared ASVs were observed in the rhizosphere and berries, as explained in Fig. 1. According to the Pearson correlation coefficient used for species clustering, several notable species were observed among our treatment and control plants, as shown in Fig. 5.

Plant development is linked with microbial progression. Studies have shown that phyllosphere [21] and rhizosphere bacterial and fungal populations of a wide variety of plants change according to plant developmental stages in Arabidopsis, Medicago, wheat, maize, pea, and sugar beet [62–65]. Here, in our study, the veraison stage was the most crucial, as reported [5]. The veraison stage scripts the commencement of the ripening process, and in grapevine phenology, this is a critical phase for metabolism and growth. Moreover, berries soften when pectin and cellulose are broken down, acidity is reduced, and sugar increases while anthocyanin synthesis and accumulation take place. All these features contribute to a more favourable microbial colonization environment.

Our findings reveal that the assemblage of fungal communities in the grapevine organs occurs in a deterministic manner according to the developmental stage. Here, the core microbial taxa abundant in the grapevine are worth highlighting the stable, essential component of the mycobial community, as only a few dominate the ecosystem [22, 66]. *Fusarium, Ilyonectria, Cladosporium and Aspergillus* remained core taxa among all the sampling groups at every phenological stage with variable abundance. The nonconformity indicates that the plant has ecological filters that favour some species while rejecting others [67, 68]. The primary taxa identified here are found in varying abundance throughout environments, with some species limited to a single plant organ.

The rare taxa, which are limited to a specific organ or phenological stage observed in the community analysis, can also be a cause of temporal transitions in microbial communities, existing as a reservoir that can quickly adapt to environmental changes, for example, and thus contribute to ecosystem resilience and stability [69, 70].

In the rhizosphere soil of treated plants, we obtained significant results for *Thermomyces. spp* at full bloom stage. These compounds are reported for the secretion of different enzymes [71] and used as industrial biocatalysts [72]. White roots resided with *Zopfiella spp.,* extracted from the cork of wine bottles made from cork oak trees [73]. We observed root colonization with some taxa described as pathogenic (*Ilyonectria, Campylocarpon* and *Rhizoctonia*) and saprophytic (*Ceratobasidium* and *Mycosphaerella*). Saprophytic fungi, in an opportunistic manner, colonize the root zone and cause deterioration of the products of root exudates, while necrotrophic pathogens could also break down root cell walls to gain access to the endorhiza [19]. made similar observations, with both saprophytic and phototrophic fungi colonizing the rhizocompartments of wine grapes in Spain. The minute richness of mycorrhizae (Rhizophagus, Glomus) in our dataset was observed, partly due to the ITS markers used for this analysis, as the small subunit rRNA region is commonly referred to in the characterization of arbuscular mycorrhizal fungal communities. The leaves of the treated plants at the veraison and maturity stages were encumbered, and different species of *Tausonia* spp. were found in the leaves of treated plants at the maturity stage. These compounds are reported to be responsible for the production of secondary metabolites named acebutolol (treatment of hypertension), epothilone D (antineoplastic agent) and isopentenyl adenosine (promotes cell division) [74]. Similarly, the flower and berry fungal species clustered in the heat map were significant and diverse. Many taxa remained unclassified, thus rendering the notion that metagenomic sequences are still underrepresented in the available databases [75].

Species-identified fungal clades according to LefSe show that the number of biomarkers regresses as we go on discovering from soil to berry. We obtained 108 different taxa in the soil, 31 in white roots, 30 in leaves, and only 5 in berries, as shown in Fig. 6. *Cutaneotrichosporon spp*. were recorded in the leaves of the treated plant at the veraison stage, were also isolated from the flower surface and were observed to have the capacity to generate higher saturated and monounsaturated fatty acid concentrations. The most influential oleic acid is thus a promising candidate for biodiesel development [76].

*C. oleaginosus* is a preferred whole-cell biocatalyst capable of producing high levels of single-cell oils from cost-effective biomass hydrolysates to metabolize a broad spectrum of monosaccharides and clashes with fermentation inhibitors [77]. OPLS-DA only enhances the efficiency of the classification in cases in which individual classes show divergence in variation within the class. The key benefit of the OPLS-DA approach lies in the fact that the generated model is more transparent because the predictive variation can be distinguished from nonpredictive (orthogonal) variation by OPLS-DA. Here, it is depicted that the variation is at maximum at the earlier stages than at the later stages and is dependent on the time scale deviation. Soil and berry were more diverse than the roots and leaves.

Various plant organs respond to environmental fluctuations in different behaviours via diverse or habitat-specific core taxa closely correlated with different environmental matrices. Increased reproductive growth and decreased vegetative growth are linked to root zone restriction. Root zone restriction eventually alters the typical plant functionalities, i.e., decreased the photosynthetic rate, chlorophyll content, and carotenoid concentration and reduced nitrate and nitrite reductase activities. Despite these anomalies in the root-zone-restricted plants, we still obtained superior quality fruit with higher total soluble (TSS) content in the presence of fungal communities in this study, while enlarged berries, increased anthocyanin content, increased volatile compounds and early berry ripening occurred through increasing abscisic acid, as reported in [29, 33, 78–80], which would eventually affect the association between grapevine and fungal communities.

## Materials and methods

### Study site

In the fruiting season of 2019, the three-year-old grapevine cultivar ‘Muscat Hamburg’ (*Vitis Vinifera* L.) plants were used for sampling from a vineyard of the School of Agriculture and Biology of Shanghai Jiao Tong University (Shanghai, China 31°11′N, 121° 29′E). Two cultivation methods, root-zone restriction as treatment (referred to as T) and conventional field cultivation as control (referred to as C), were evaluated. Root-restricted plants were grown in a cylindrical plastic container at 60 cm × 90 cm (height×width). Control plants were grown on raised beds (50 cm height). The ratio of the growing medium was used as soil:sand:organic fertilizer (5:1:1) for both types of plants. Vine spacing was 2 m, and row spacing distance was 3 m in north-south oriented rows. Both vines (root-restricted and standard field-grown) were maintained under the same soil, climate, and management conditions. Nutrition, irrigation, and pruning were carried out as previously mentioned by [32].

### Measurement of physiological parameters and total soluble sugars

Mature fully expanded two apical leaves from each vine and three treatment and control plants were used to record chlorophyll content with a portable meter (SPAD-502PLUS, Minolta, Tokyo, Japan).

After peeling the berries, the grains and flesh were sieved out and put in centrifuge tubes and centrifuged for 10 min at 4 °C and 10,000 rpm. A Brix meter was used to calculate the total soluble solids (TSS) content of the supernatant, ATAGO Master-M, Tokyo, Japan [81].

For the treatment and control, three vines for each were selected. Ten shoots per vine were randomly designated to measure shoot length, making a total of thirty biological replicates at each phenological stage. Trunk and cane diameters were also measured with a digital Vernier calliper. The sampling time of these physiological parameters was the same as the sampling time of the other plant organs and soil. TSS sampling was conducted at pre-veraison, veraison and maturity. The data were analysed as a factorial experiment based on a completely randomized design with three replications. The contrast between the means was carried out by using Fisher LSD as a post hoc test at *P* < 0.05 using SPSS software.

### Soil sampling

The rhizosphere and four different plant compartments, white roots, leaves, flowers and berries, were sampled at the full bloom stage (May), veraison (June), and maturity (August), 2019. Plants were chosen to represent the homogeneity (exposure to existing sunlight and wind) and location in the vineyard (start to the end of the row).

Roots were acquired from a depth of 15-20 cm for rhizosphere soil fraction in root-zone restricted and 20-30 cm in control plants. Sampled roots with rhizosphere soil particles attached were placed in sterile tubes containing 9 mL of physiological solution (9 g/L NaCl). The tubes were vortexed for 5 min to detach the soil particles and then centrifuged at 4000 rpm for 5 min. The supernatant was discarded, and the remaining soil fraction was used to represent the rhizosphere fraction. White roots were hunted from a depth of 15-20 cm for treated plants, while we dug 20-30 cm deep for the roots of control plants, and the roots were picked manually with sterile gloves.

Healthy leaves of identical sizes with no apparent symptoms of disease were collected. Different bunches containing undamaged berries were chosen as described by [82]. All samples were taken in triplicate, retained in sterile bags immediately, stored in liquid nitrogen in the field, and later transferred to − 80 °C until DNA isolation.

### DNA extraction and amplicon sequencing

Total genomic DNA was extracted using the OMEGA Soil DNA Kit (D5625-01) (Omega Bio-Tek, Norcross, GA, USA) and stored at − 20 °C before further analysis. A NanoDrop ND-1000 spectrophotometer (Thermo Fisher Scientific, Waltham, MA, USA) and agarose gel electrophoresis were used to determine the quantity and quality of extracted DNA, respectively. PCR amplification of the fungal ITS1 region was achieved using the forward and reverse primers ITS5F and ITS2R, respectively [83]. Barcodes unique to the 7-bp sample were unified into primers for multiplex sequencing. Vazyme VAHTSTM DNA Clean Beads (Vazyme, Nanjing, China) were used to purify the PCR amplicons and quantified using the Quant-iT PicoGreen dsDNA Assay Kit (Invitrogen, Carlsbad, CA, USA). Amplicons were pooled in equal quantities after the individual quantification process, and paired-end 2 * 250 bp sequencing was performed at Shanghai Personal Biotechnology Co., Ltd. (Shanghai, China) using the Illumina MiSeq platform with MiSeq Reagent Kit v3.

### Bioinformatics analysis

Computational analyses were performed on metagenome sequences with QIIME2 2019.4 [34]. Specifically, using the demux plugin, raw sequence data were demultiplexed following primer cutting with the cutadapt plugin [84]. The DADA2 plugin was used for quality filtering, denoising, merging, and chimaera removal of the acquired sequences [85]. MAFFT was used to align with nonsingleton amplicon sequence variants (ASVs) [86]. Classify-sklearn with a naïve Bayes classifier was used for assigning the taxonomy to ASVs [87] against the Unite database release 8.0 with 99% ASV reference sequences [88]. Moreover, a phylogenetic tree was constructed using fastTree-2 [89].

QIIME2 and R packages (v3.2.0) were used to conduct data analyses. The taxonomic composition was analysed by the QIIME taxa bar plot command for each plant organ and rhizosphere at the phylum and genus levels. ASV-level alpha diversity indices were determined using the ASV (amplicon sequence variant) table in QIIME2 using the Chao1 richness estimator and Shannon diversity index and visualized as box plots. To compare the richness and evenness of ASVs among samples, we created ranked abundance curves.

Bray-Curtis distance metrics were used for beta diversity insights to examine the structural heterogeneity of fungal species across samples [90]. Then, the cells were visualized via principal coordinate analysis (PCoA) and the unweighted pair-group method with arithmetic means (UPGMA) hierarchical clustering [91]. PERMANOVA (permutational multivariate analysis of variance) evaluated the importance of differentiating the fungal structure between groups [92]. The petal diagram was generated by the R package (v3.2.0) (VennDiagram) to visualize the shared and unique ASVs between samples or groups,

Centred on the abundance of ASVs, we used the abundance data of the top 50 genera in average abundance to further compare the taxa distribution at the genus level in sample groups and drew a heat map. By using the default parameters, linear discriminant analysis impact size (LefSe) was performed as a supervised model to detect differentially abundant taxa across plant organs and the rhizosphere. OPLS-DA (orthogonal partial least squares discriminant analysis) was also implemented to expose the variation in fungal communities between groups using the ‘muma’ R package [93].

## Conclusions

The present study revealed that fungal richness and diversity possess an idiosyncratic trend of intra-vineyard variability at grapevine phenological stages. At the veraison stage, grapevine habitats differed the most. High-throughput sequencing of the fungal ITS1 region showed that root-zone restriction strongly impacts fungal communities associated with different grapevine habitats. The interaction of microbes was presumably well observed by measuring the TSS of the berries, and the relative abundance of fungal species as berries of root-zone restricted plants had a peak value of TSS along with the highest relative abundance of *Aspergillus* species. This study underlies a new path of investigation of the role of *Aspergillus* in fruit quality enhancement.

## Supplementary Information

**Additional file 1.** Petal map diagram showing the unique ASVs and the core ASVs among different sample groups. Rhizosphere soil (a), white roots (b), leaves (c), berry (d).

**Additional file 2.** Length distribution of the high-quality sequences contained in all samples. The number of BP is on the x-axis, and the number of sequences obtained is on the Y-axis.

**Additional file 3.** Venn diagram showing the sharing of the core ASVs obtained from the flower petal Fig. 1.

**Additional file 4.** Total ASV count per sample. The abscissa is arranged according to the sample name, while the ordinate is the number of ASVs in each sample classified to any classification level.

**Additional file 5.** Histogram showing the relative abundance of the top 20 fungal phyla recorded from the different groups of Rhizosphere A, White roots B, Leaves C and berry D at three phenological stages of Fullbloom FB, Veraison V and Maturity M.

**Additional file 6.** Rank abundance curve, arranges the ASVs in each sample according to their abundance along the abscissa, and uses the respective abundance values as the ordinate. The flatness of the broken line reflects the uniformity of the community composition for rhizosphere (a), white roots (b), leaves (c), and flowers/berries (d).

**Additional file 7.** Species accumulation curves are used to measure and predict the increase in species richness in a community as the sample size increases, and the sample size is sufficient to estimate the community abundance for rhizosphere (a), white roots (b), leaves (c), and flowers/berries (d).

**Additional file 8.** UPGMA clustering. Hierarchical clustering is often used to display the similarity between samples in the form of a hierarchical tree, and the clustering effect is measured by the branch length of the clustering tree. The shorter the branch length between samples, the more similar the two samples, rhizosphere (a), white roots (b), leaves (c), and flower/berry (d).

**Additional file 9.** Details of the taxa shown in the cladogram for rhizosphere (a), white roots (b), leaves (c), and flowers/berries (d).

**Additional file 10.** Statistics of received sequence volume per sample.

**Additional file 11.** Statistical table of annotation results of species taxonomy per sample.

**Additional file 12.** Table for Brix values of TSS of berries (Control and treated plant) at each phenological stage.

**Additional file 13.** The detailed table for Permanova (Adonis) for rhizosphere and plant organs.

## Data Availability

The raw sequence read datasets generated and analysed during the current study are available at the National Centre for Biotechnology Information Sequence Read Archive under the bio project PRJNA717898. https://www.ncbi.nlm.nih.gov/bioproject/?term=PRJNA717898.
