# Supplementary figures and images for: Comparative fungal diversity and dynamics in plant compartments at different developmental stages under root-zone restricted grapevines
Source: BMC Microbiol. 2021 Nov 16;21:317. doi: 10.1186/s12866-021-02376-y (PMC8594160; doi:10.1186/s12866-021-02376-y)

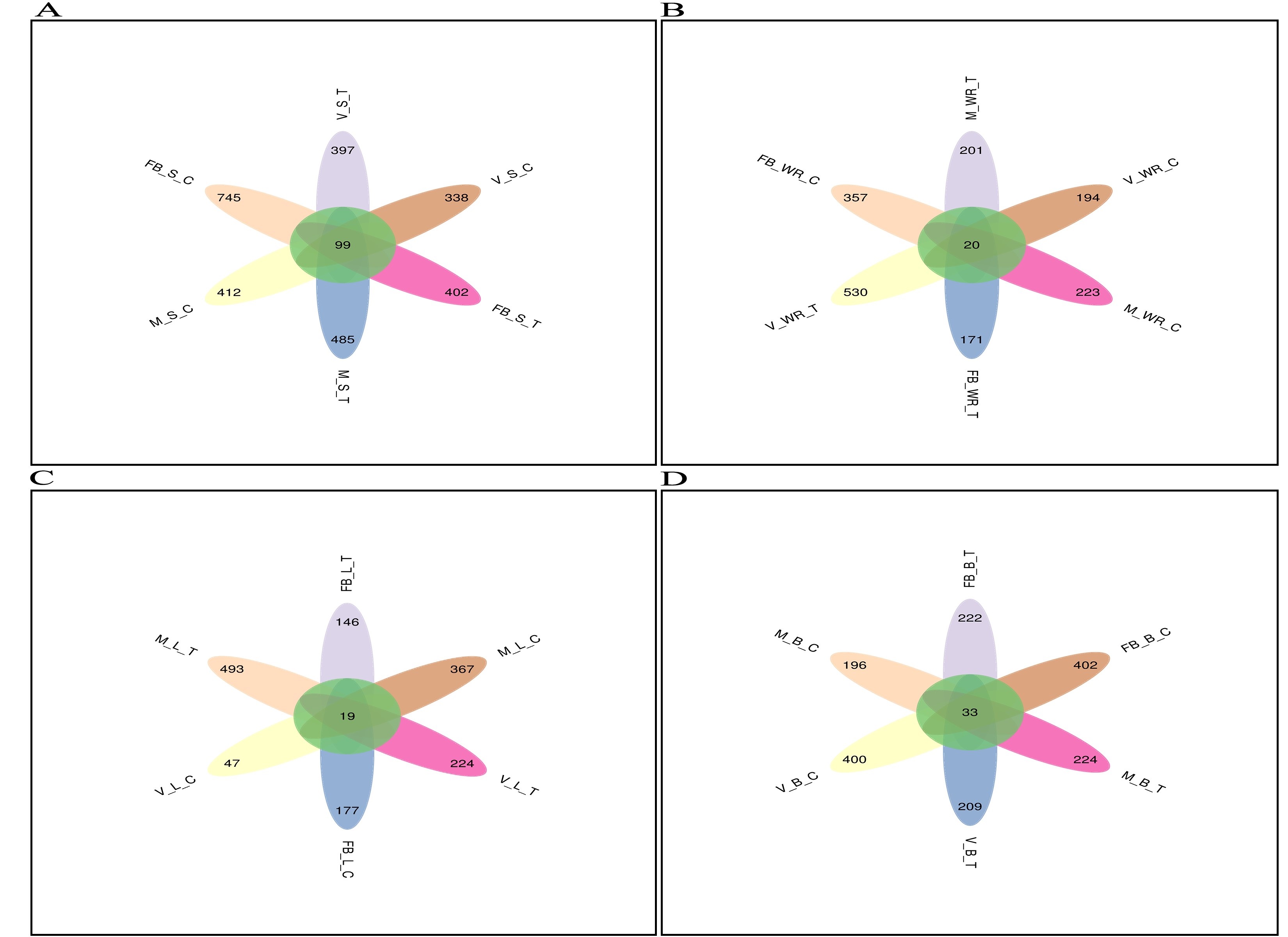

Supplement: Supplementary file 1 — Additional file 1. Petal map diagram showing the unique ASVs and the core ASVs among different sample groups. Rhizosphere soil (a), white roots (b), leaves (c), berry (d). [file 12866_2021_2376_MOESM1_ESM.jpg]

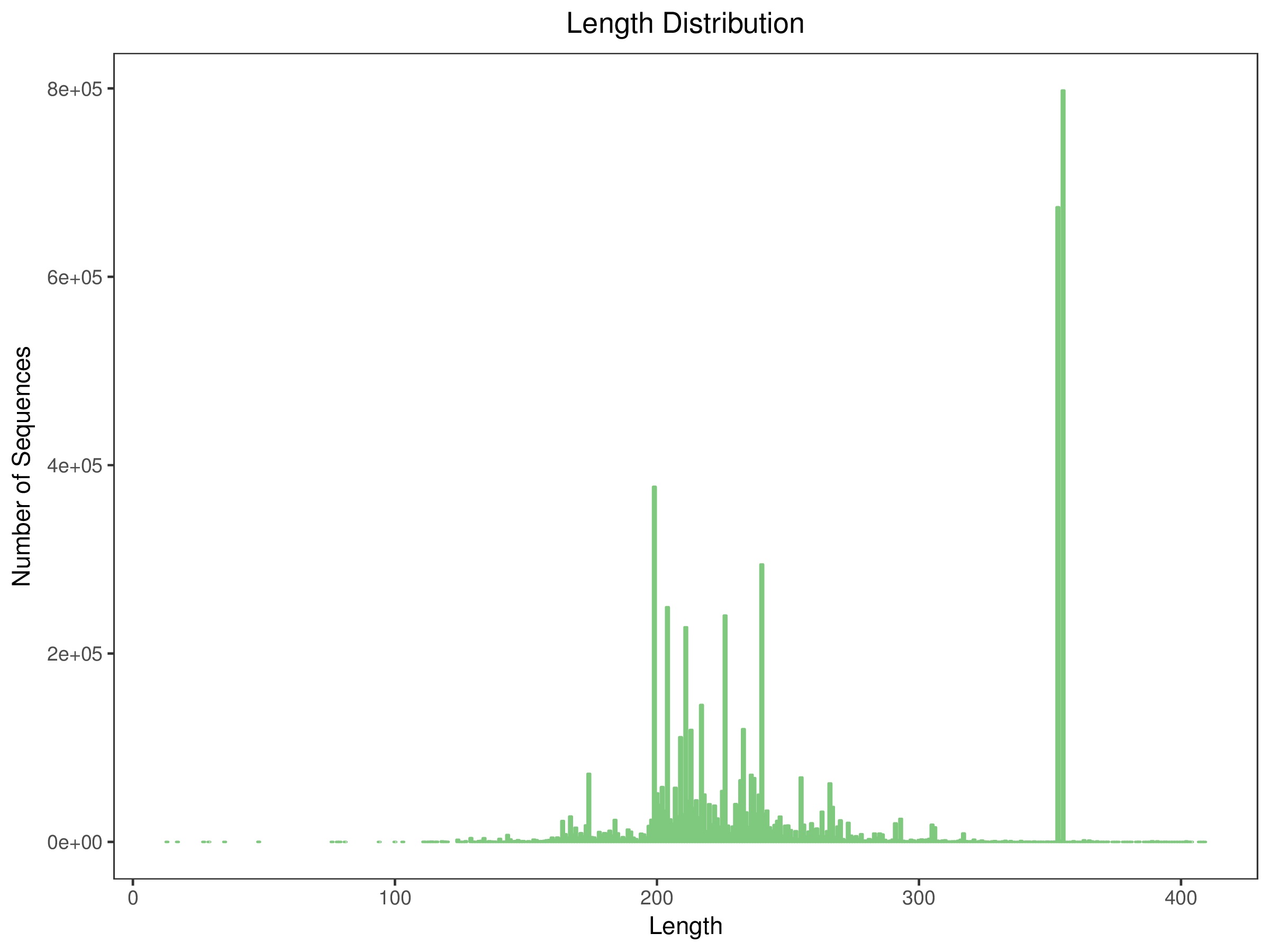

Supplement: Supplementary file 2 — Additional file 2. Length distribution of the high-quality sequences contained in all samples. The number of BP is on the x-axis, and the number of sequences obtained is on the Y-axis. [file 12866_2021_2376_MOESM2_ESM.jpg]

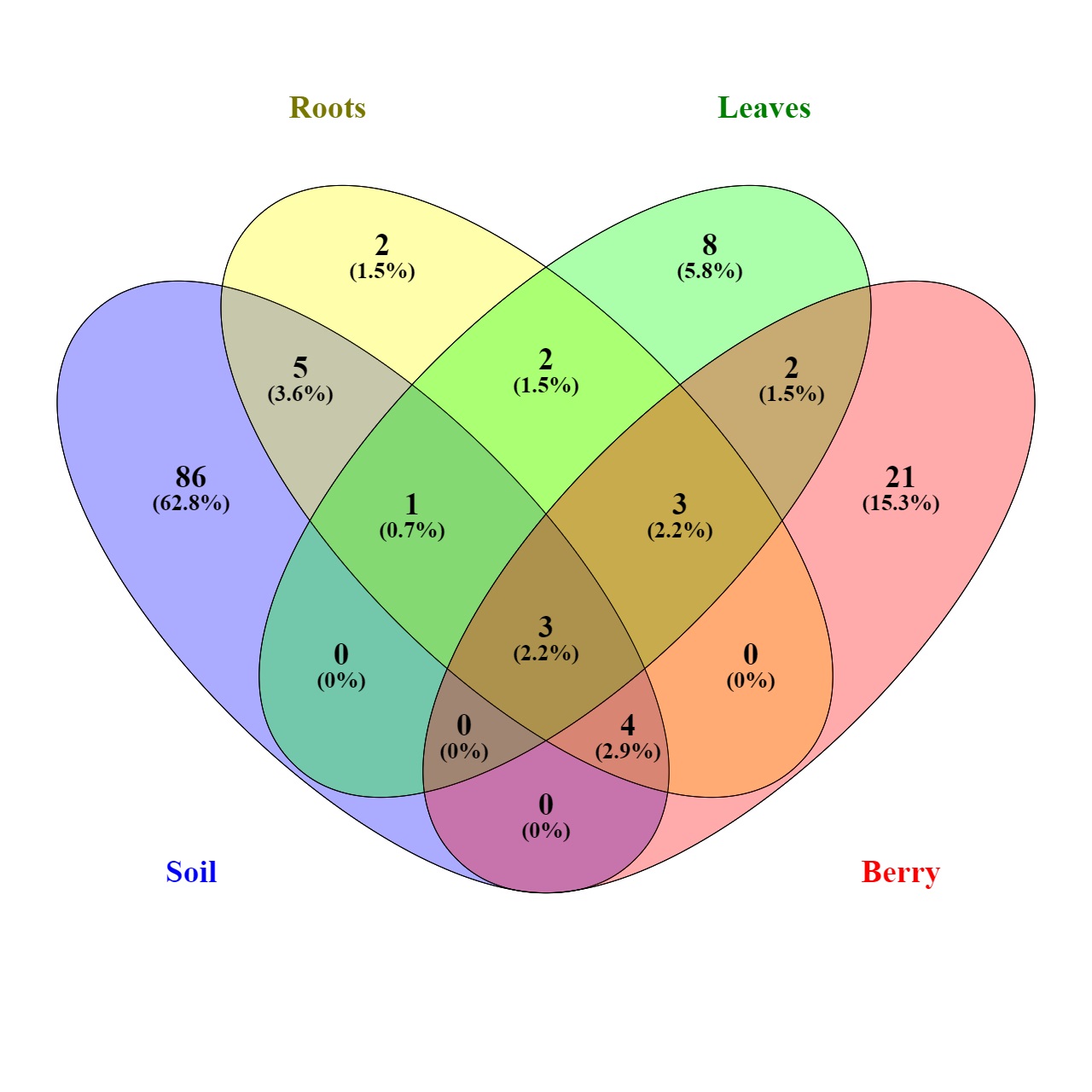

Supplement: Supplementary file 3 — Additional file 3. Venn diagram showing the sharing of the core ASVs obtained from the flower petal Fig. 1. [file 12866_2021_2376_MOESM3_ESM.jpg]

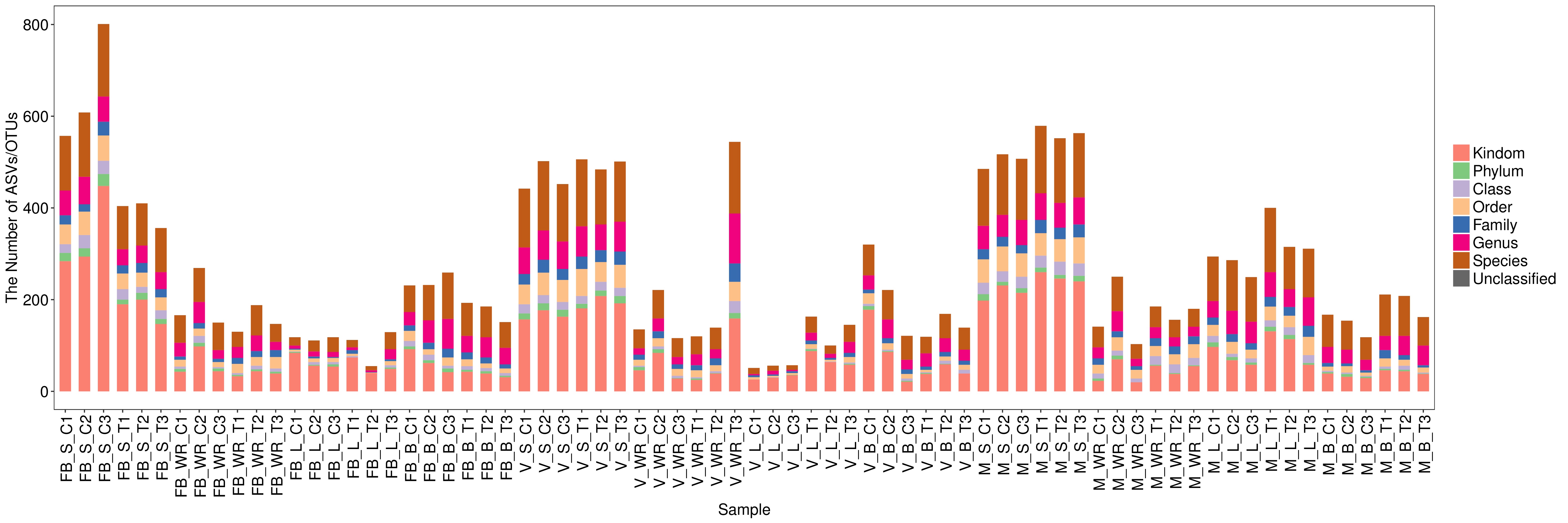

Supplement: Supplementary file 4 — Additional file 4. Total ASV count per sample. The abscissa is arranged according to the sample name, while the ordinate is the number of ASVs in each sample classified to any classification level. [file 12866_2021_2376_MOESM4_ESM.jpg]

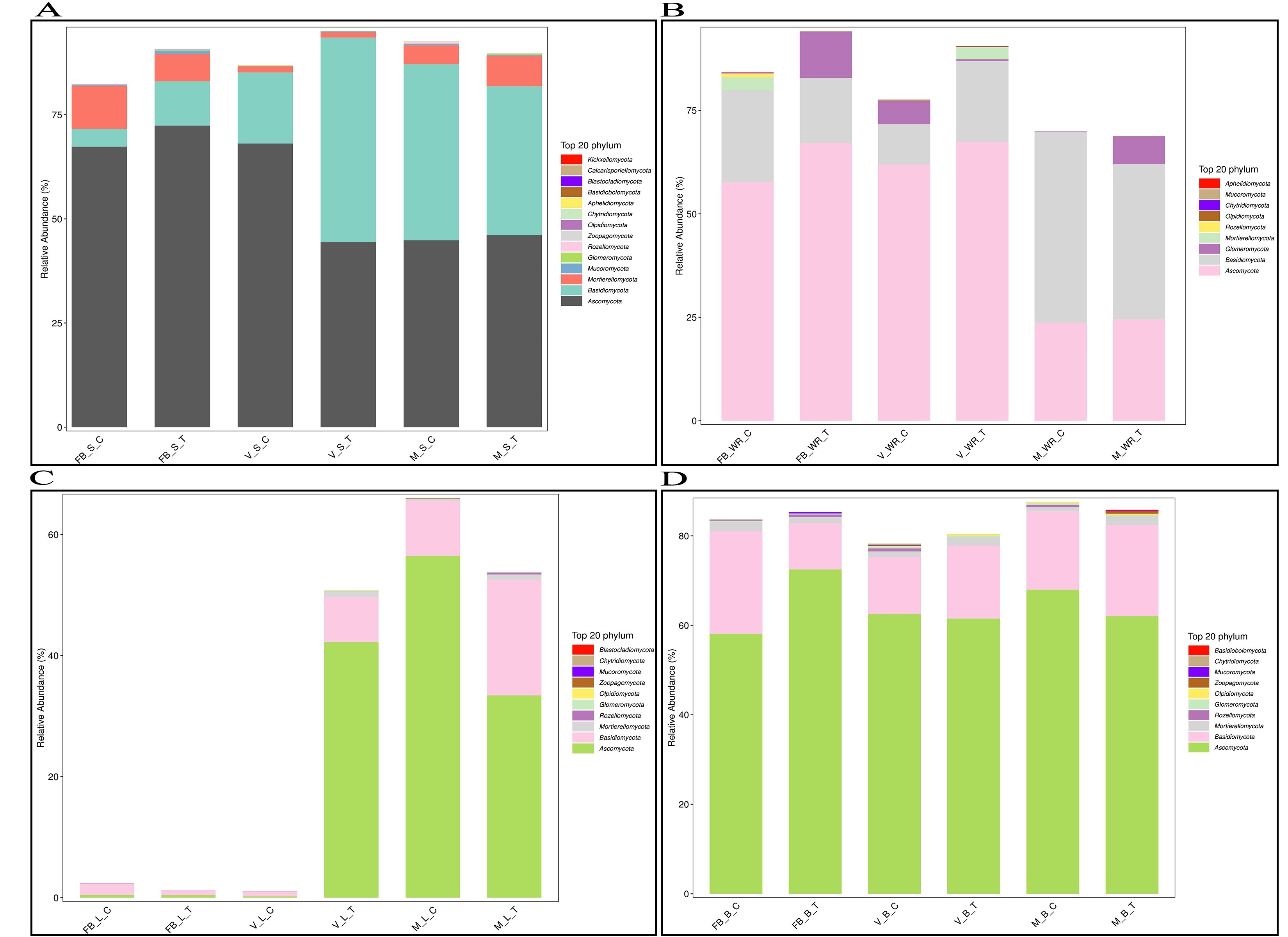

Supplement: Supplementary file 5 — Additional file 5. Histogram showing the relative abundance of the top 20 fungal phyla recorded from the different groups of Rhizosphere A, White roots B, Leaves C and berry D at three phenological stages of Fullbloom FB, Veraison V and Maturity M. [file 12866_2021_2376_MOESM5_ESM.jpg]

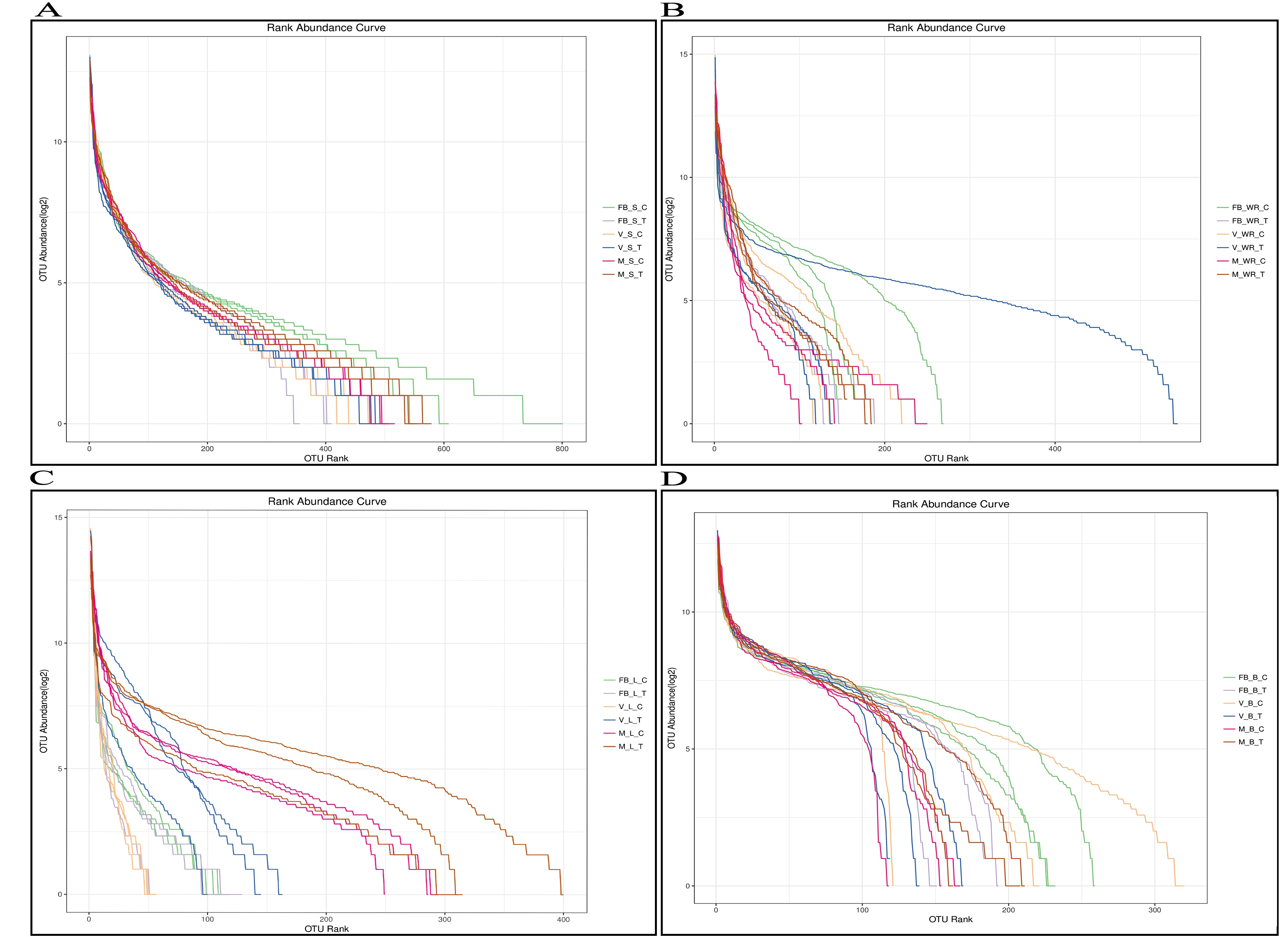

Supplement: Supplementary file 6 — Additional file 6. Rank abundance curve, arranges the ASVs in each sample according to their abundance along the abscissa, and uses the respective abundance values as the ordinate. The flatness of the broken line reflects the uniformity of the community composition for rhizosphere (a), white roots (b), leaves (c), and flowers/berries (d). [file 12866_2021_2376_MOESM6_ESM.jpg]

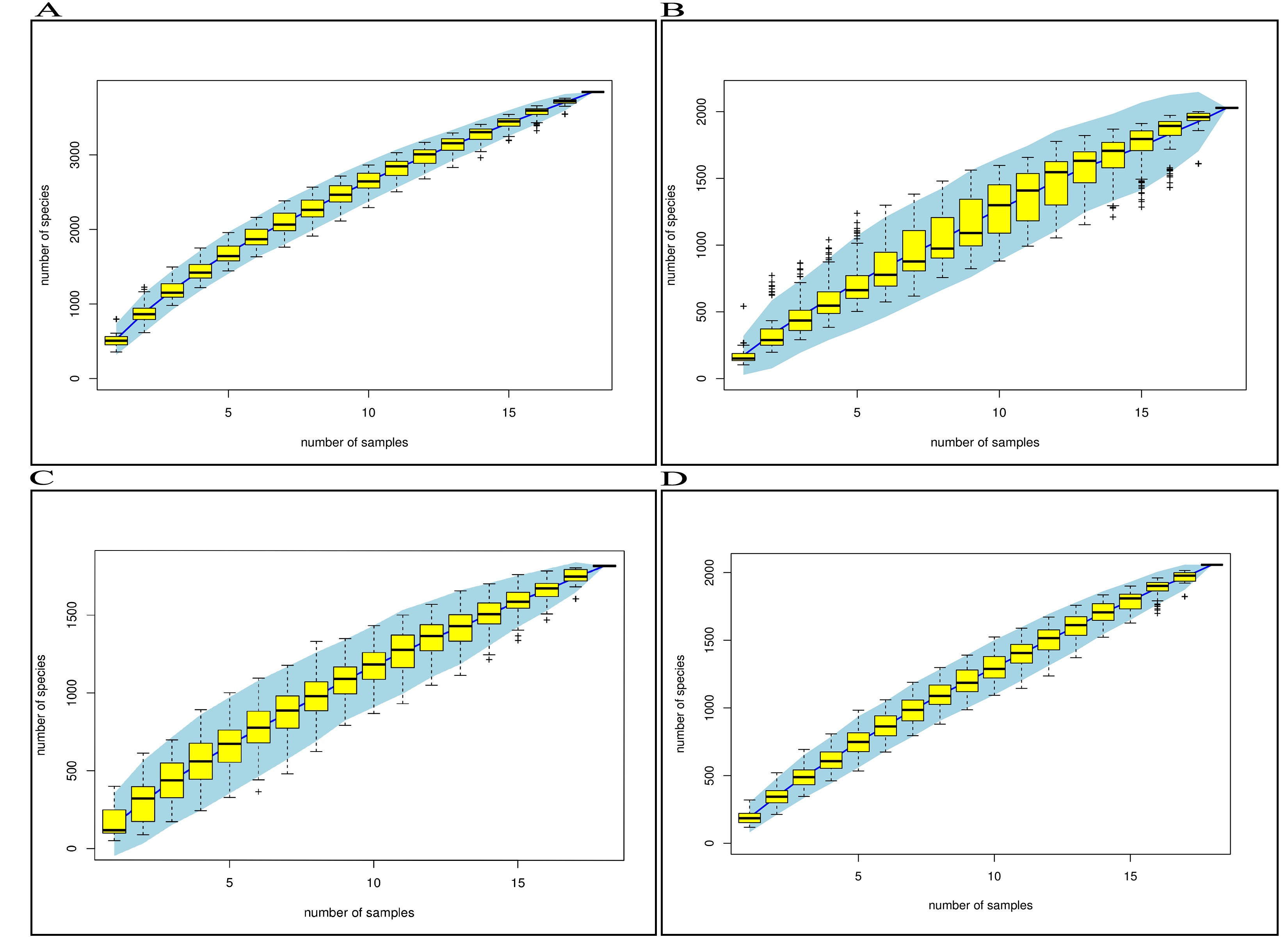

Supplement: Supplementary file 7 — Additional file 7. Species accumulation curves are used to measure and predict the increase in species richness in a community as the sample size increases, and the sample size is sufficient to estimate the community abundance for rhizosphere (a), white roots (b), leaves (c), and flowers/berries (d). [file 12866_2021_2376_MOESM7_ESM.jpg]

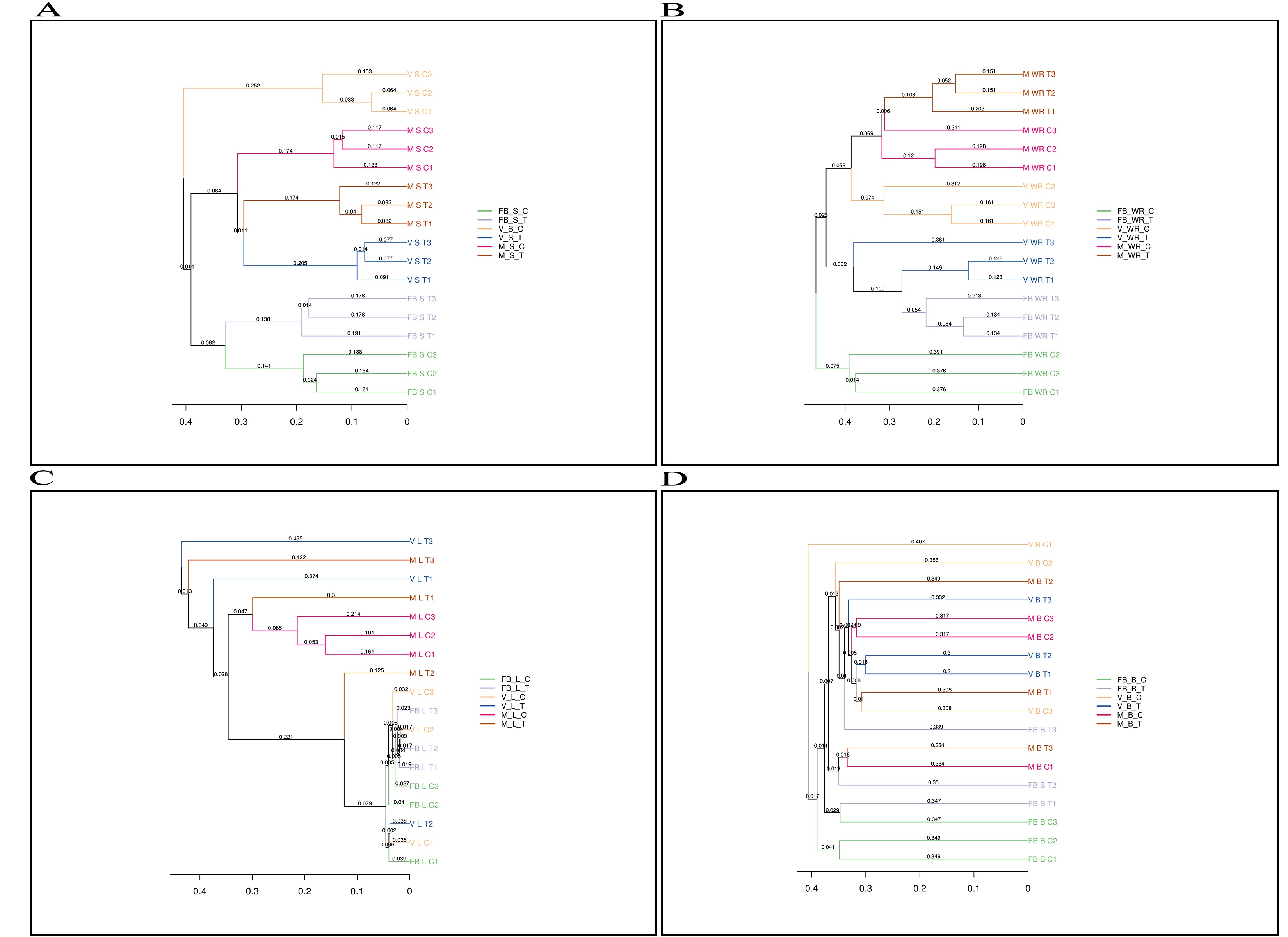

Supplement: Supplementary file 8 — Additional file 8. UPGMA clustering. Hierarchical clustering is often used to display the similarity between samples in the form of a hierarchical tree, and the clustering effect is measured by the branch length of the clustering tree. The shorter the branch length between samples, the more similar the two samples, rhizosphere (a), white roots (b), leaves (c), and flower/berry (d). [file 12866_2021_2376_MOESM8_ESM.jpg]

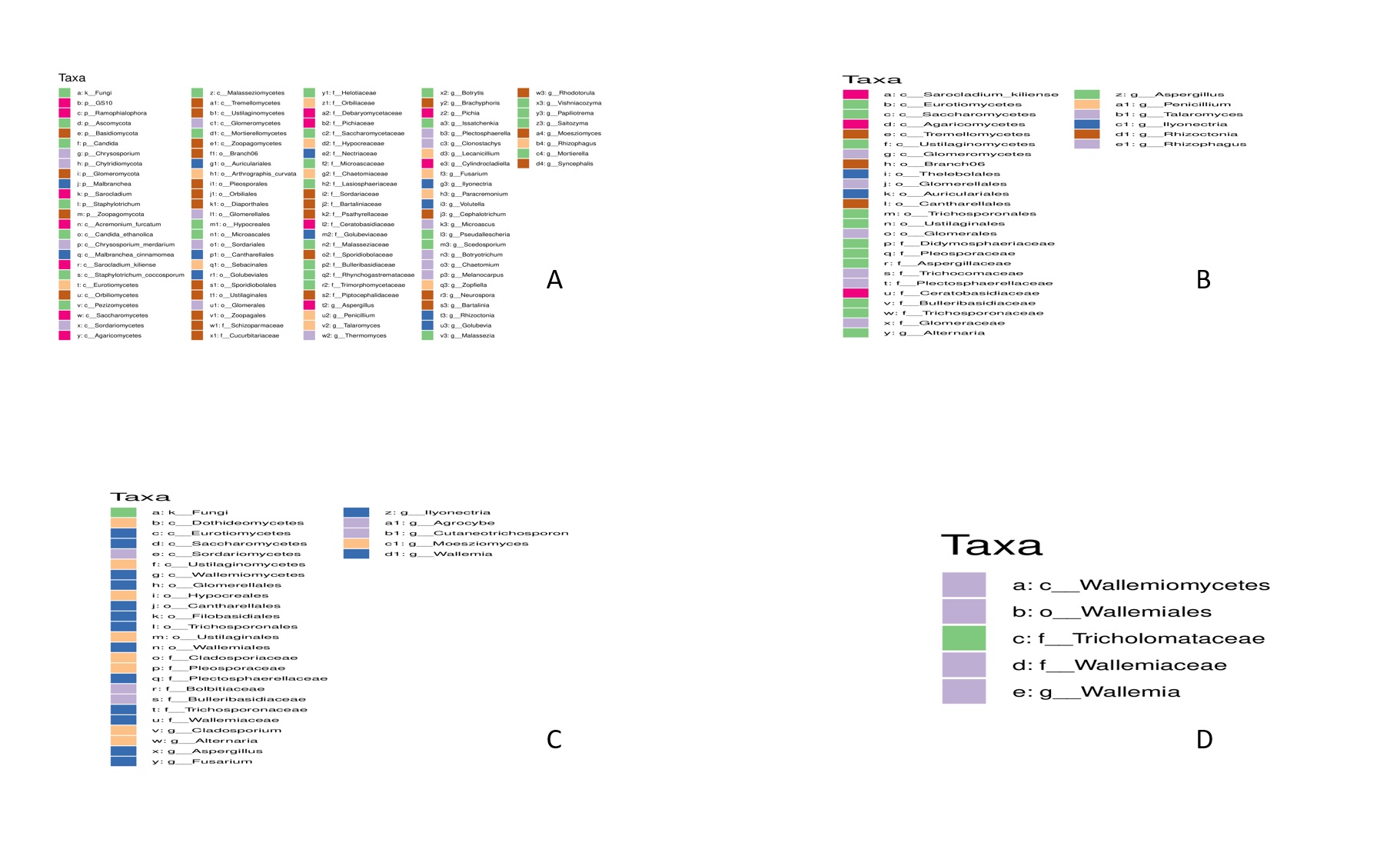

Supplement: Supplementary file 9 — Additional file 9. Details of the taxa shown in the cladogram for rhizosphere (a), white roots (b), leaves (c), and flowers/berries (d). [file 12866_2021_2376_MOESM9_ESM.jpg]
